# Supplementary figures and images for: Herpetogaster collinsi from the Cambrian of China elucidates the dispersal and palaeogeographic distribution of early deuterostomes and the origin of the ambulacrarian larva
Source: PeerJ. 2023 Nov 7;11:e16385. doi: 10.7717/peerj.16385 (PMC10637255; doi:10.7717/peerj.16385)

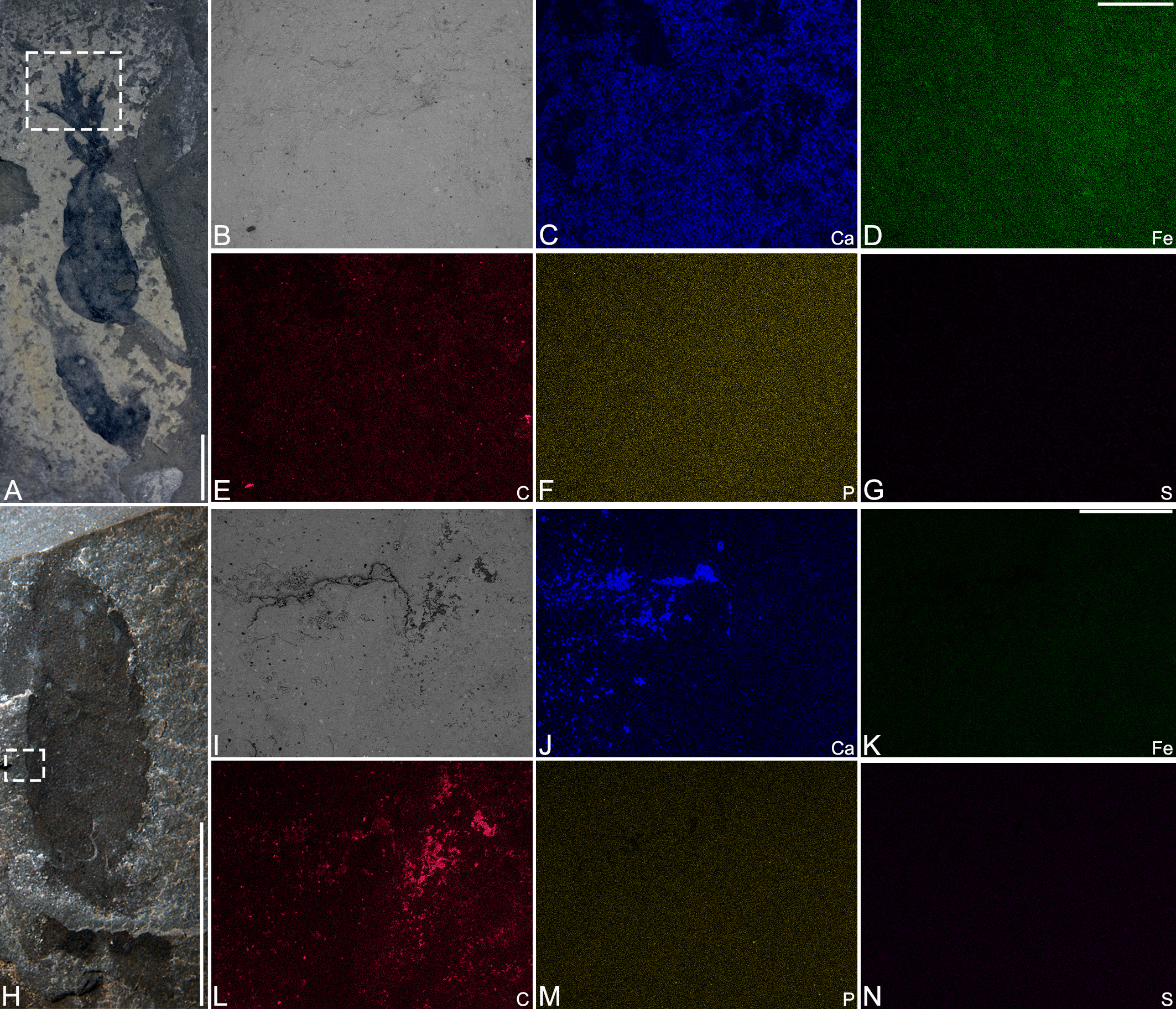

Supplement: Supplemental Information 2 — (A–G) YKLP 14573. (A) Picture of the specimen indicating the analyzed area (dashed rectangle). (B) Detailed view of the analyzed area. (C–G) SEM-EDS elemental maps of Ca, Fe, C, P, S, respectively. (H–N) YKLP 14583. (H) Picture of the specimen indicating the analyzed area (dashed rectangle). (I) Detailed view of the analyzed area. (J–N) SEM-EDS elemental maps of Ca, Fe, C, P, S, respectively. Scale bars: (A, H) 5mm; (C–G, J–N) 1 mm. [file peerj-11-16385-s002.jpg]
